# Shifts in Plant Community Assembly Processes across Growth Forms along a Habitat Severity Gradient: A Test of the Plant Functional Trait Approach

**DOI:** 10.3389/fpls.2018.00180

**Published:** 2018-02-15

**Authors:** Jinshi Xu, Yongfu Chai, Mao Wang, Han Dang, Yaoxin Guo, Yu Chen, Chenguang Zhang, Ting Li, Lixia Zhang, Ming Yue

**Affiliations:** ^1^Key Laboratory of Resource Biology and Biotechnology in Western China, Ministry of Education, Northwest University, Xi'an, China; ^2^School of Life Sciences, Northwest University, Xi'an, China; ^3^College of Grassland and Environment Sciences, Xinjiang Agricultural University, Urumchi, China

**Keywords:** functional traits, habitat severity, community assembly, climate change, functional structure, trait-trait relationships, woody species, herbaceous species

## Abstract

Species respond to changes in their environments. A core goal in ecology is to understand the process of plant community assembly in response to a changing climate. Examining the performance of functional traits and trait-based assembly patterns across species among different growth forms is a useful way to explore the assembly process. In this study, we constructed a habitat severity gradient including several environment factors along a 2300 m wide elevational range at Taibai Mountain, central China. Then we assessed the shift on functional trait values and community assembly patterns along this gradient across species among different growth forms. We found that (1) although habitat-severity values closely covaried with elevation in this study, an examined communities along a habitat severity gradient might reveal community dynamics and species responses under future climate change. (2) the occurrence of trait values along the habitat severity gradient across different growth forms were similar, whereas the assembly pattern of herbaceous species was inconsistent with the community and woody species. (3) the trait-trait relationships of herbaceous species were dissimilar to those of the community and woody species. These results suggest that (1) community would re-assemble along habitat severity gradient through environmental filtering, regardless of any growth forms and that (2) different growth forms' species exhibiting similar trait values' shift but different trait-trait relationship by different trait combinations.

## Introduction

The climate acts as a filter for the species pool on a regional scale (Southwood, 1988), as it shifts the interactions of plant species (Chapin et al., 1998), as well as the community assembly process. Plant functional traits and their value's distribution patterns have become proxies for examining the plant community assembly process (Grabherr et al., 1994; Díaz et al., 1999, 2007; Mcgill et al., 2006; Vittoz et al., 2008; Walther et al., 2009; Mason and de Bello, 2013; Yablon, 2013). Such approaches might reveal species responses to climate change (Weiher and Keddy, 1995; Woodward and Cramer, 1996; Díaz and Cabido, 1997; Cornwell and Ackerly, 2009; Götzenberger et al., 2012; Mason et al., 2012; Spasojevic and Suding, 2012; May et al., 2013).

Plant functional traits are the outcome of a history of species adaptation (Southwood, 1988). They represent the primary strategy that plants utilize to adapt to a changing environment (Lamanna et al., 2014). Plants respond to variable habitat conditions by adjusting their metabolism (Pappas et al., 2016) and performance (Keddy, 1992). Meanwhile, habitats filter species according their particular combination of traits (Keddy, 1992). Thus, plant functional traits, especially “response traits” which are measurable characteristics of plants, are assumed to reflect a plant's response to changes in its habitat (McIntyre et al., 1999; Lavorel et al., 2007; Meng et al., 2009; Borchardt et al., 2013). Response traits also provide information on the physiological adaptations of vegetation to various environmental gradients (McIntyre et al., 1999; de Bello et al., 2005; May et al., 2013; Purcell, 2016). These processes are believed to shape the range of functional trait values within communities (Cornwell and Ackerly, 2009) through habitat filtering or interspecific competition. Convergence of a trait value suggests co-occurring species often appeared in similar abiotic conditions, leading to habitat filtering (Grime, 2006; Cornwell and Ackerly, 2009). In contrast, interspecific competition is expected to exclude species with high trait similarity, resulting in trait divergence (MacArthur and Wilson, 1967; Weiher and Keddy, 1998; Stubbs and Wilson, 2004; Kraft et al., 2009). These functional traits distribution patterns can be described by using standardized effect sizes (SES) of traits. The SES of traits observed value to null expectation value (Kraft et al., 2009; Kraft and Ackerly, 2010).

As communities' functional trait values would change under the assembly process, species' trait value would change within community. It is necessary to examine the relationship between functional trait values and the associated biotic and abiotic conditions where the plant community is established to predict assembly patterns with climate change. Plant functional traits are assumed to be adaptively differentiated with habitats differing in some key factors (e.g., disturbance) or resource availability (Brouillette et al., 2014). Previous studies have focused on the trait-habitat relationships along various gradients in different regions and scales (Díaz and Cabido, 1997; Fonseca et al., 2000; Westoby and Wright, 2002; Wright et al., 2004; Fynn and Kirkman, 2005; Lambrecht and Dawson, 2007; Cornwell and Ackerly, 2009; Qi et al., 2009; Maharjan et al., 2011; Violle et al., 2011; Lawson and Weir, 2014). However, since plant functional traits vary among life forms (e.g., woody vs. herbaceous plants in one community, Meng et al., 2015), the concept of plant growth form is important for community dynamics (Meng et al., 2015). Nevertheless, previous studies have largely focused on woody species or particular species in forest communities (Cunningham et al., 1999; Fonseca et al., 2000; Qi et al., 2009), rather than herbaceous species (Oyarzabal et al., 2008). Studies on functional trait values might reveal whether woody or herbaceous species respond differently to environmental gradients (Yablon, 2013). Moreover, analyzing the assembly process in woody/herbaceous level and in community level separately is a useful way to detect the actual community assembly mechanisms comprehensively. These studies may be helpful to reveal the most focused ecological question of plant community, while they are seldom to be involved.

Examining different growth forms' responses seems necessary to explore the community assembly which respond to changes in environmental gradients (Keddy, 1992; Westoby, 1998). However, there are few studies examining the response of functional trait/functional trait distribution to the combination of these environment factors (Weiher and Keddy, 1995; de Bello et al., 2006) or plant functional traits (Smith and Wilson, 1994). We developed a habitat severity matrix representing the level of environmental stress in the habitat. We measured functional traits (leaf morphologic traits, leaf chemometrical traits, plant height, and seed mass) of each species in the communities based on the intrinsic dimensions theory (Laughlin, 2014; Laughlin and Messier, 2015). Additionally, we assessed the functional traits values and their distributions changes along the environmental severity gradient across varying levels (community, woody species, and herbaceous species) at Taibai Mountain, central China.

We expected that (1) the community assembly pattern would show convergence in more stressful habitat, and (2) economic spectrum-related functional trait-trait relationships and functional trait distributions would demonstrate different patterns across different growth forms.

## Materials and methods

### Study site

The study was carried on the Taibai Mountain Nature Reserve, central China, located on 33°59′45″N−34°05′12″N, 107°41′18″E−107°48′22″E. This temperate zone is expected to permit a wider range of trait combinations than the tropical zone (Lamanna et al., 2014) since there are more heterogenous micro niches. The elevations of the reserve region vary from 940 to 3,767 m. In the present study, we selected the core area of forest which elevations range from 1,140 to 3,481 m. Such a wide-range altitudinal gradient provides opportunities to study various responses of plant species to changes in habitat conditions (Cordell and Handley, 1999). The climate of the study site is dominated by continental monsoon, mean annual temperature (MAT) varies from 0.9 to 12.3°C related to elevation, and annual precipitation is 640–1,000 mm (Tang and Fang, 2004). Forest coverage is over 82%, with high species diversity. In our study region, 389 woody species potentially exist which are recorded in the literature; the vegetation distributes along zonal zone in Taibai Mountain (Xu et al., 2017). The zonal vegetation of the Taibai Mountain region is highly heterogeneous in the warm temperate-zone deciduous broad-leaved forest and coniferous forest resulting from differences in hydrothermal conditions along such a wide-range altitudinal gradient (Zhu, 1981).

### Plot setup and sampling

Along the altitudinal gradient, we established 39 plots; the area of each plot is 20 × 30 m. We selected more than 3 plots per 200 m elevation range, which were set as far as possible in order to represent the whole study region integrally. These selected plots commonly have different topographic factors or species composition in order to maximize variation in the environmental factors (sampling maps see Appendix Figure 1). Main field work was conducted in July 2014.

We collected soil samples dug from the 10–20-cm layer below ground surface and litter at four corners of the plots. We collected soil samples within same date for making sure the weather condition's consistency of samples. Soil samples were sealed in plastic bags, and they were naturally dried with 3 weeks for experimental analysis. In addition, we also recorded the habitat information, including elevation, location (by HOLUX EZ-Tour GPS recorder, HOLUX Technology Inc.), topographic slope (by a specific compass), and canopy coverage.

All species within each plot were identified, the abundance and coverage of the species in each plot were documented. The abundance of woody species (DBH > 8 cm) was counted as the number of stems. The abundance of herbaceous species was calculated by its relative coverage via assignment: We established eight 0.3 × 0.3 m quadrats within each plot for recording the abundance and coverage of each herbaceous species. Based on these data, we estimated the total abundance of herbaceous species within each plot (Marteinsdóttir and Eriksson, 2014). We recorded plant height of each woody species individual. For individuals <2 m height, height was measured using a tape, while individuals > 2 m height were measured using a height indicator; thereafter, the maximum plant height of each woody species was determined. For herbaceous species, the tallest individual was chosen to measure plant height. Because leaves are most exposed to habitat conditions and the changes in their traits have been interpreted as adaptations to specific environments (Fahn and Cutler, 1992), we collected 18–20 fully expanded sun-exposed leaves at top of crown from various directions of each mature species individuals within each plot. For each species, we selected at least five individuals if possible. The leaf samples were preserved in moist filter paper until analysis (Pérez-Harguindeguy et al., 2013).

### Traits measuring

We measured eight functional traits. Apart from the highest plant height (Hmax) mentioned above, we measured leaf morphologic and chemometrical traits by using leaf samples we collected. Leaf area (LA) is a trait determined by gradients in available moisture and temperature (Meng et al., 2009), Specific leaf area (SLA) is key trait reflecting species resource acquisition strategies (Cheng et al., 2016). Leaf dry matter content (LDMC) is another basic important leaf morphologic traits linking to habitat conditions (Yan et al., 2012). These leaf morphologic traits were measured following standard methods (Cornelissen et al., 2003). We also measured leaf chemometrical traits. Leaf nitrogen content (LNC), leaf carbon content (LCC) were measured by elemental analyzer (EA3000, EuroVector Inc.), and the leaf carbon-nitrogen ratio (C:N) was calculated afterwards. We also obtained seed mass (SM) data by weighing seed specimen preserved in the specimen museum. Missing data on seed mass of some species were compensated by literature review or website information (http://data.kew.org/sid/sidsearch.html). It is fine to assess the community assembly patterns for using these traits, as it would minimize the number of traits but maximize the number of dimensions (Laughlin, 2014).

Although trait values are often weighted by its relative abundance (Garnier et al., 2004; Violle et al., 2007) which calculated by its abundance divided by the sum of total abundance numbers within a community. In our study, there were species belonging different growth forms, relative abundance may not reflect the information of their actual biomass. Here we weighted the trait values of each species by its importance value (IV). The importance value of each species in each community was calculated as the sum of its relative abundance, relative height, and relative coverage and then divided by 3 (Xu et al., 2017). Note that the importance value of each species was calculated separately for all species combined (community level), only canopy species (woody species level), and only undergrowth herbaceous species (herbaceous species level). We calculated the weighted mean trait values of community, woody species and herbaceous species of each plot afterwards (method see Díaz et al., 2007).

### Environmental factors

Traits are related to temperature, light, moisture availability, soil pH and nutrients. (Landolt et al., 2010). Here we developed a “habitat-severity value” as an agency of all these factors. We measured the soil pH by the pH indicator (PB-100, Sartorius Inc.). Soil fertility factors nitrate nitrogen content (NN), ammonium nitrogen content (AN), total nitrogen content (TN), and rapidly available phosphorus (RAP) were analyzed by discontinuous chemical analyzer (CleverChem 2000, Dechem-Tech Inc.). Soil water content (SWC) which calculated by the ratio of water mass to total soil mass was measured as an indicator of moisture availability. We used woody species canopy coverage degree (WCD) as habitat cover. Furthermore, topographical slope was measured in our study, as it showed correlation with species leaf traits and height (Ackerly and Cornwell, 2007). MAT and air relative humidity (RH) were calculated by empirical equations adapted to Taibai Mountain (Tang and Fang, 2004):

(1) $$\begin{array}{lll} \text{MAT} & = & -0.0049\;\times \text{ ALT }+\;17.9\;\left({\text{r}}^{2}=0.99,\text{ P}<0.001\right) \end{array}$$

(2) $$\begin{array}{lll} \text{RH} & = & 0.4\;\times \;10^{-6}\;\times \;{\left(\text{ALT}\right)}^{2}-0.0153\;\times \text{ ALT}\\ +\;83.7\;\left({\text{r}}^{2}=0.95,\text{ P}<0.01\right) \end{array}$$

In the equations, ALT indicates elevation of plots.

### Constructing habitat severity matrix

As there are numerous traits, we used principal component analysis (PCA) of all these traits for dimensionality reduction (Shipley, 2015). The PC1 axis of community weighted mean trait values captured 55.56% of the total variance of traits, while the PC1 axes of woody species and herbaceous species weighted mean trait values captured 52.09 and 36.89%, respectively. The PC1 axes of community, woody species and herbaceous species seemed to have statistical correlation with almost all the traits (Appendix Table 1). We used the PC1 scores of three levels as the trait variables to perform variance decomposition. We input PC1 scores as dependent variables, while input environmental factors as independent variables for variance decomposition, which was performed in “hier.part” package of R 3.1.1 program. Variance decomposition (Pappas et al., 2016) was used to identify the relative roles in variance of traits in community level and in woody or herbaceous species level. We obtained the relative roles of factors in every habitat for trait variations in community, woody species and herbaceous species level respectively. The results of variance decomposition were in form as percentage (Appendix Table 2).

Thereafter, we constructed the habitat-severity values for above three levels. All the habitat factors were normalized to [0, 1], the more stressful for plant growth, the value is more nearby 1. Therefore, we used these scores as the habitat-severity values, in other words, as habitat severity gradient.

### Data analysis

We performed linear regression between weighted mean traits values and habitat-severity values in community, woody species and herbaceous species levels. In addition, we assessed the functional trait distribution of each plot in community, woody species and herbaceous species level as well by using SES of traits. The SES which describes functional trait distribution is the degree of discrepancy of trait observed value to null expectation value (Cornwell and Ackerly, 2009; Kraft et al., 2009; Kraft and Ackerly, 2010). Before calculating SES, mean functional distance (MFD) within a community should be calculated (Webb, 2000). MFD describes the mean difference between two species.

$$\begin{array}{l} SES\;=\;\frac{MFD_{observed-MFD_{randomized}}}{sdMFD_{randomized}} \end{array}$$

**Figure 1:**
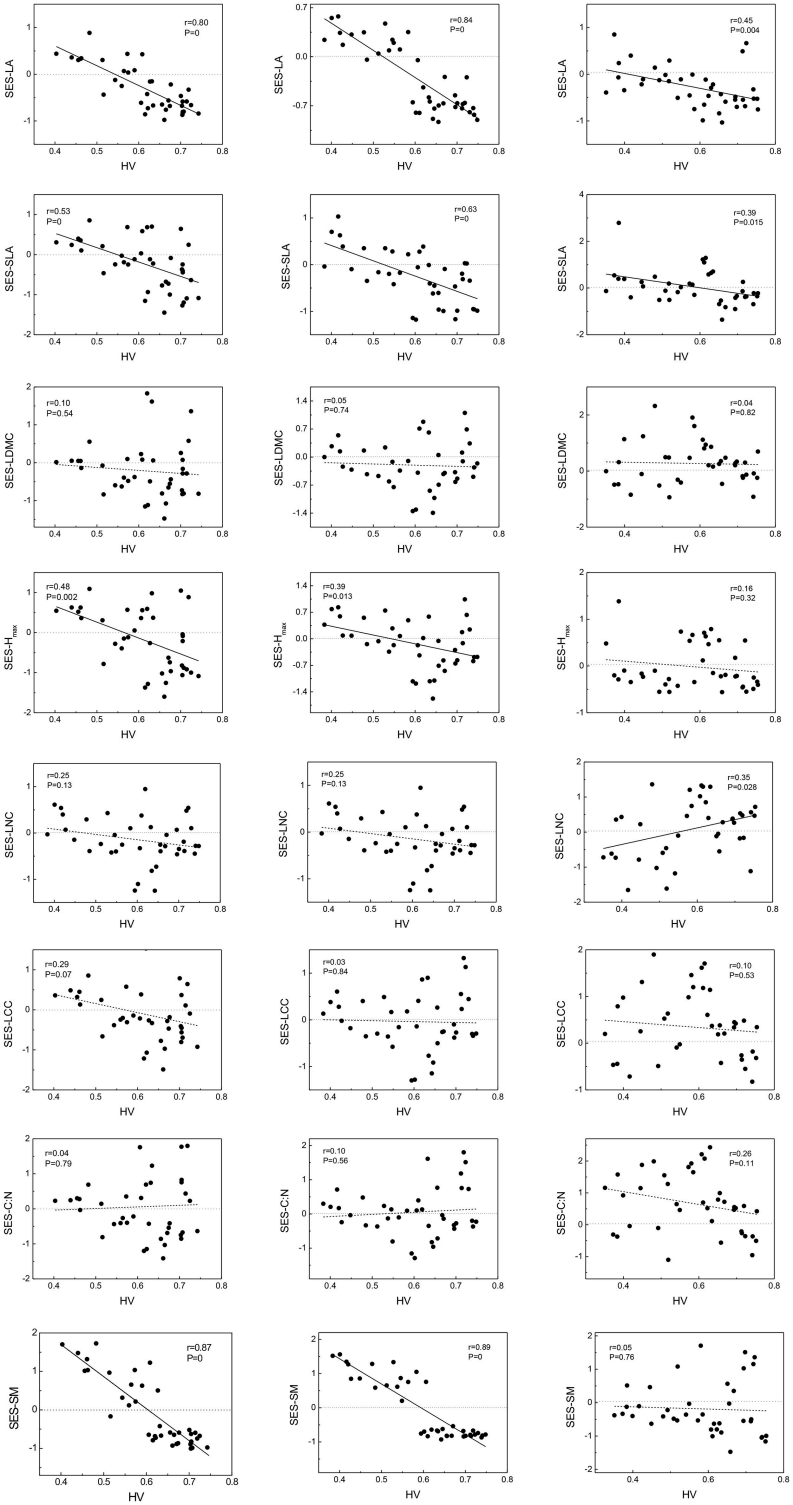
Standardized effect sizes (SES) of traits vary along habitat-severity values (HV) gradient. Graphs on left column represent woody species level, graphs on right column represent herbaceous species level, and graphs on central column represent community level. Solid straight lines in graphs mean fit lines which *p* < 0.05; dash lines in graphs mean fit lines which *p* > 0.05. Dot lines locate on 0-level represent patterns of null expectation. LA, leaf area; SLA, specific leaf area; LDMC, leaf dry mass content; Hmax, the max plant height; LNC, leaf Nitrogen content; LCC, leaf Carbon content; C:N, leaf Carbon-Nitrogen ratio; SM, seed mass, same in below.

In the equation, *MFD*_*observed*_ is actual MFD which calculated by observed functional traits values; *MFD*_*randomized*_ is calculated by null model approach which is run in R 3.1.1 program for 999 times; sd means standard deviation. If SES < 0, means functional structure convergence; in contrast, SES > 0 indicates functional structure divergence. Owing to convergence or divergence of functional structures mirrored community assembly processes, we could assess performances of woody or herbaceous species comparing to community total species along habitat severity gradient and understand more detailed assembly processes (Figure 1).

We also showed the trait-trait relationships among above levels in order to determine differentiation of trait-response among different levels. We tested the Pearson correlation coefficient of each trait in community, wood species, and herbaceous level (Tables 2, 3) by using the weighted values, and then compared with each other so that we could find out patterns which were consistent or not among different components in common community. As we found our habitat-severity values was dominated by MAT and RH which calculated by elevation (Appendix Table 2, Appendix Table 3), we also compared the Pearson correlation coefficient of elevations/habitat-severity values to weighted mean functional trait values in above three levels for assessing the habitat severity matrix (Table 1).

**Table 1:** The correlation coefficient of mean weighted trait values and elevation/habitat-severity values (HV).

**Trait**	**HV**	**Elevation**	**Trait**	**HV**	**Elevation**	**Trait**	**HV**	**Elevation**
C-LA	**−0.797**	**−0.822**	W-LA	**−0.819**	**−0.839**	H-LA	**−0.393**	**−0.459**
C-SLA	**−0.513**	**−0.699**	W-SLA	**−0.294**	**−0.589**	H-SLA	**−0.675**	**−0.734**
C-LDMC	**0.464**	**0.534**	W-LDMC	**0.336**	**0.482**	H-LDMC	**0.479**	**0.429**
C-Hmax	**−0.692**	**−0.622**	W-Hmax	**−0.685**	**−0.597**	H-Hmax	**−0.360**	**−0.342**
C-LCC	**0.652**	**0.672**	W-LCC	**0.438**	**0.464**	H-LCC	**0.594**	**0.592**
C-LNC	−0.113	−0.143	W-LNC	−0.143	−0.287	H-LNC	**0.392**	**0.505**
C-C:N	**0.460**	**0.444**	W-C:N	**0.463**	**0.536**	H-C:N	−0.114	−0.206
C-SM	**0.888**	**−0.911**	W-SM	**−0.854**	**−0.901**	H-SM	**−0.333**	**-0.442**

*C-, W-, and H- trait represents mean weighted trait values of community, woody species, and herbaceous species. The Pearson correlation coefficient which has statistical significance is highlighted in bold*.

**Table 2:** The trait-trait relationship semi-matrix of woody species.

	**LA**	**SLA**	**LDMC**	**Hmax**	**LCC**	**LNC**	**C:N**	**SM**
LA		**0.522**	**−0.553**	**0.633**	**−0.651**	0.135	**−0.426**	**0.811**
SLA	**0.454**		**−0.658**	**0.386**	**−0.479**	**0.475**	**−0.520**	**0.572**
LDMC	**−0.458**	**−0.653**		**−0.420**	**0.557**	**−0.355**	**0.490**	**−0.511**
Hmax	**0.629**	0.155	−0.225		**−0.632**	0.179	**−0.477**	**0.758**
LCC	**−0.497**	**−0.397**	**0.555**	−0.301		0.025	**0.321**	**−0.720**
LNC	0.191	**0.563**	**−0.355**	0.043	0.155		**−0.814**	0.100
C:N	**−0.466**	**−0.563**	**0.436**	−0.289	0.152	**−0.820**		**−0.490**
s SM	**0.841**	**0.455**	**−0.445**	**0.742**	**−0.466**	0.199	**−0.518**	

*Mind that the semi-matrix at top right corner is the trait-trait relationship within community level. The Pearson correlation coefficient which has statistical significance is highlighted in bold*.

**Table 3:** The trait-trait relationship semi-matrix of herbaceous species.

	**LA**	**SLA**	**LDMC**	**Hmax**	**LCC**	**LNC**	**C:N**	**SM**
LA		**0.522**	**−0.553**	**0.633**	**−0.651**	0.135	**−0.426**	**0.811**
SLA	**0.340**		**−0.658**	**0.386**	**−0.479**	**0.475**	**−0.520**	**0.572**
LDMC	**−0.375**	**−0.620**		**−0.420**	**0.557**	**−0.355**	**0.490**	**−0.511**
Hmax	−0.061	0.216	0.029		**−0.632**	0.179	**−0.477**	**0.758**
LCC	−0.259	**−0.520**	**0.542**	−0.113		0.025	**0.321**	**−0.720**
LNC	−0.024	−0.152	−0.297	**−0.388**	0.035		**−0.814**	0.100
C:N	−0.074	−0.090	**0.460**	**0.335**	**0.343**	**−0.874**		**−0.490**
SM	0.278	**0.435**	**−0.346**	0.112	−0.265	−0.117	−0.058	

*Mind that the semi-matrix at top right corner is the trait-trait relationship within community level. The Pearson correlation coefficient which has statistical significance is highlighted in bold*.

## Results

### Performances of habitat-severity values

Habitat severity values (HV) were dominated by MAT and RH, and MAT and RH were calculated using the elevations of plots. The correlation analysis between HV and elevation confirmed the notion that other environmental factors might correlate with elevation, even across three different levels (Appendix Table 3). We found nearly equal correlations between HV and traits and elevation and traits, regardless of level (Table 1).

### Functional trait distributions vary along habitat severity gradient: different patterns across different growth forms

Functional trait distribution of community and woody species exhibited almost complete similarity (no significant difference by U-test). Without exception, the functional distributions of LA, SLA, Hmax, and SM across community and woody species levels showed convergent tendencies with higher habitat severity. For herbaceous species, although there were convergent tendencies on LA and SLA along habitat severity, the inflection points of divergence-convergence were inconsistent with community and woody species. The SES of herbaceous LNC showed a divergent tendency at higher habitat severity. The SES of other traits did not show significant correlation with the habitat severity gradient (Figure 1).

### Functional traits vary along habitat severity gradient

**Figure 2:**
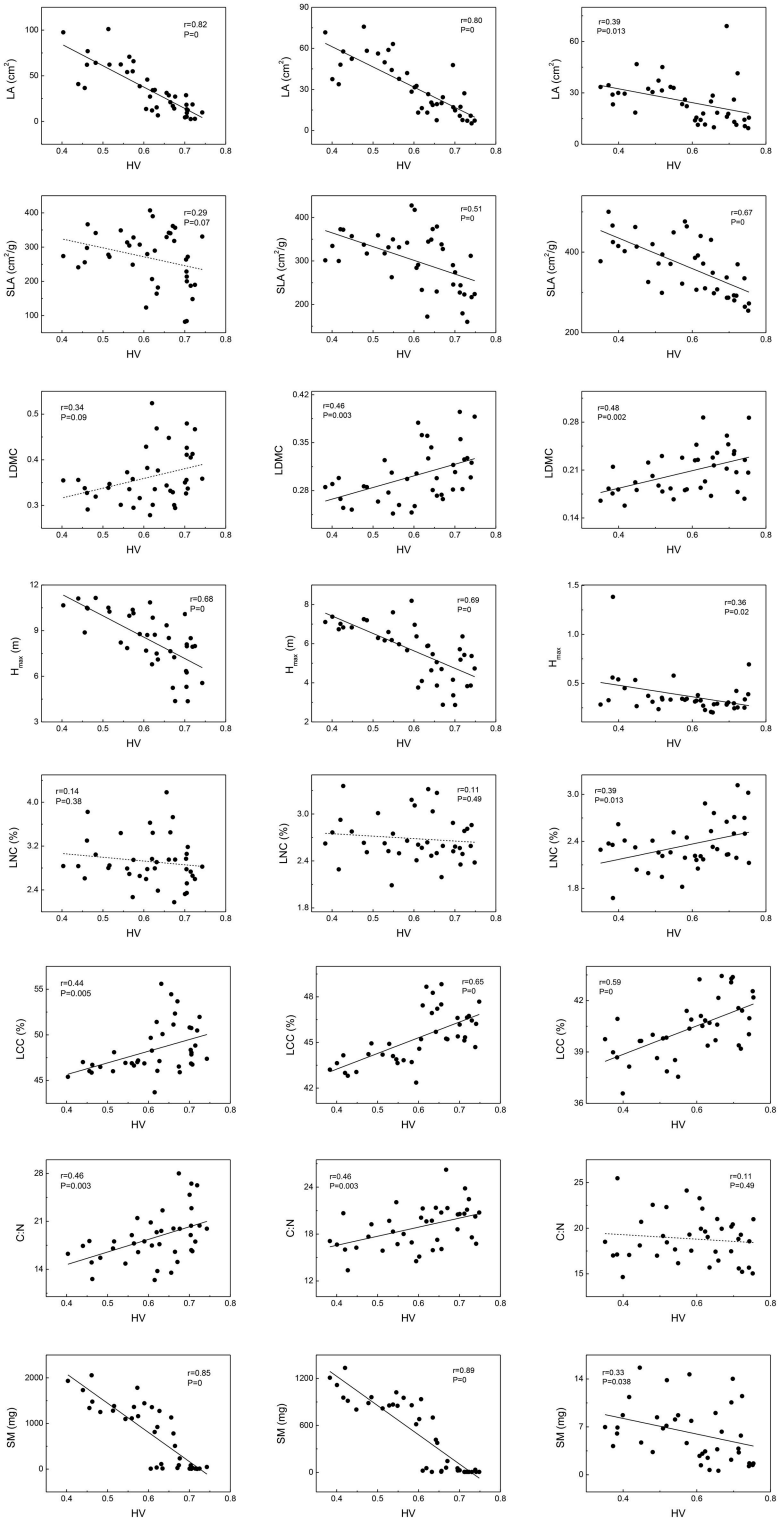
Mean weighted trait values vary along habitat-severity values (HV) gradient. Graphs on left column represent woody species level, graphs on right column represent herbaceous species level, and graphs on central column represent community level. Solid straight lines in graphs mean fit lines which *p* < 0.05; dash lines in graphs mean fit lines which *p* > 0.05.

Variation of traits among the community, woody species, and herbaceous species along habitat severity gradients displayed similar patterns to each other (Figure 2). For Hmax and SM, patterns among different levels were almost consistent; Hmax and SM decreased along the habitat severity gradient, though the slope of fit lines in herbaceous species were relatively flat. Chemometrical traits among the community, woody species, and herbaceous species exhibited interesting patterns; the LCC of these levels were consistent with each other (increasing with habitat severity), while LNC demonstrated a significant increase in only herbaceous species. Due to the varying performances of LNC, C:N showed similar patterns along habitat seventy. For leaf morphological traits, the LA of community, woody species, and herbaceous species exhibited decreased tendencies along habitat severity gradient. SLA and LDMC of woody species did not demonstrate significant relationships to habitat severity; however, community and herbaceous species exhibited increased patterns.

### Trait-trait relationships show different patterns across different growth forms

The Pearson correlation semi-matrices of woody species and herbaceous species were compared with the semi-matrix of community (Tables 2, 3). Trait-trait relationships of community and woody species were similar, except for very few trait-trait relationships, such as the relationships which involving Hmax. However, there were relatively different trait-trait patterns between the community and herbaceous species. Although the patterns of traits in community and herbaceous species levels along habitat severity gradient shifted in a similar manner, the relationships between traits each other of community was dissimilar to those relationships of herbaceous species.

## Discussion

### Habitat severity gradient applications in plant response to more stressful condition

The plant responses to environmental gradients seems to be a result of habitat severity, which links to the environmental filtering effect (Díaz et al., 1999). In the past, ecological studies elevations that varied in temperature, moisture availability, and other environment factors (Guittar et al., 2016) for community assembly processes. We developed habitat-severity values (HV) to assess the severity of habitat conditions. Our HV is strongly correlated with elevation (Appendix Table 3), and the correlations between HV and traits are similar to those between elevation and traits; this suggests that elevation might replace habitat severity in regions with a wide range of elevations but short geographic distances. Some studies revealed that the filtering process occurred at higher elevations (Pottier et al., 2012; Hulshof et al., 2013), similar to the pattern observed at high habitat severity in our study. However, even in regions without an obvious gradient, we suggest applying this framework to explore the shift in functional traits and their distributions. This framework could reflect plant response to more stressful conditions in changing world.

### Assembly processes is different across different growth forms

The process of community assembly is related to environmental conditions. Generally speaking, plant traits tend to be clustered under stressful conditions, suggesting the occurrence of habitat filtering. Under less extreme environmental conditions, plant traits exhibit high differentiation due to interspecific competition (Kluge and Kessler, 2011). In our study, functional trait distributions of community and woody species were covariant in all examined traits along habitat severity gradient, whereas the functional trait distribution of herbaceous species showed dissimilar patterns. These results are consistent with other studies (Cornwell and Ackerly, 2009; Yablon, 2013) and suggest differing responses to habitat change across different growth forms. However, it should be noted that the robustness of HV for herbaceous plants is unclear, as the PC1 of herbaceous species' traits for structuring the HV only explained 35% of the total variance in traits. These results might be explained by environmental factors which were not measured in our study, such as light availability or herbivore activity. These factors might have a stronger influence on herbaceous species than on woody species, potentially resulting in inconsistency compared to the community and woody species.

### Community assembly pattern changes with habitat

The functional trait-based approach is a good approach for understanding plant community assembly patterns. Functional trait distributions change along the habitat severity gradient in this study and seems worthwhile to discuss. With increasingly stressful environmental conditions, the functional distributions of LA, SLA, Hmax, and SM of the community and woody species showed convergent patterns. In other words, abiotic filters restricted the range of viable strategies, thus creating a similar suite of traits (Woodward and Diament, 1991; Thuiller et al., 2004; de Bello et al., 2005; Fraser et al., 2016. These results suggest the filtering effect of habitat severity (Díaz et al., 1998; May et al., 2013). In fact, functional trait distributions will change along several environmental gradients which were included in our habitat severity matrix. For example, SLA is restricted with temperature (Joshi, 2013) and soil moisture (Cornwell and Ackerly, 2009); Hmax of shade-tolerant species is filtered by WCD (Cornwell and Ackerly, 2009). It would be interesting to discuss the convergent tendency of SM along the habitat severity gradient, as it seldom exhibited this pattern (Garnier and Navas, 2012). Due to the abundant species diversity in our study, highly-conserved traits—such as seed mass (Cavender-Bares et al., 2006)—would exhibit greater divergence at low to middle altitudinal regions than at higher elevations. As a result, seed mass demonstrated a convergent tendency along the habitat severity gradient, which was closely related to elevation. Therefore, the community assembly pattern would change as habitat changed.

### Functional traits vary along habitat severity gradient in similar pattern across different growth forms

Our results demonstrated similar patterns of trait-habitat relationships among community, woody species, and herbaceous species, suggesting species within a community respond to environmental conditions similarly. Leaf traits, plant height, and seed mass correlated with environment conditions (Díaz et al., 1998; Cornelissen, 1999). Habitat filtering might cause these shifts in mean weighted trait values along environmental gradients (Ackerly and Cornwell, 2007; Cornwell and Ackerly, 2009; May et al., 2013). Species located in more arid sites had significantly lower LA, confirming a functional trade-off between stress tolerance and productivity in leaves (Thuiller et al., 2004). SLA is lower at regions with low rainfall and/or temperature, owing to thicker leaves and/or denser tissues (Tranquillini, 1964; Hadley and Smith, 1986; Westoby and Wright, 2002). Such habitats lead to higher LDMC and LCC in species as defense against stressful conditions (Oyarzabal et al., 2008). Plant height was also strongly correlated with stress (usually decreased, Lamanna et al., 2014), particularly when directly related to resource competition (e.g., temperature and light availability; Schwinning and Weiner, 1998). Smaller seed mass is often observed in species from more stressful conditions (Cornelissen, 1999; Luo et al., 2014). Such adaptations might allow plants to disperse to better environmental conditions (Körner, 1999). Our findings are consistent with the previously mentioned studies. In our study, higher habitat severity values mirrored lower temperature, water availability, and poorer soil conditions. At the community level, as habitat severity increased, community mean weighted trait values of LA, SLA, Hmax, and SM decreased, as well as findings in herbaceous' level. However, SLA and LDMC of woody species did not decrease along habitat severity gradient significantly; this phenomenon might be caused by the morphological differences between broad-leaved species and needle-leaved species, which often appear at middle to high elevations (middle to high HV in this study). We also observed that LNC of herbaceous species increased with the habitat severity gradient; this is consistent with a study in high-mountain grasslands, representing a greater investment in photosynthetic nitrogen (Díaz and Cabido, 1997).

It should be noted that we only focused on interspecific trait differences, which are the primary source of variability in trait values. The traits we selected for this study often show low phenotypic plasticity (especially for leaf stoichiometry, SLA, LDMC, and seed mass).

### Trait-trait relationships of herbaceous species are unique compared to community

Analyses of intra-community trait-trait relationships can reveal the functional trade-offs operating along gradients of environmental stress. Dwyer and Laughlin (2017) proposed a novel insight that, with habitat becoming more stressful, trait covariance would become more significant. This phenomenon could be inferred from the patterns observed in functional distributions. In more stressful habitats, functional traits often show convergent patterns. Thus, two traits are more likely to exhibit significant covariance. For the community and woody species in our study, LA, SLA, Hmax, and SM showed significant tendencies to decrease along the habitat severity gradient (Figure 1). Combining the patterns of these traits along habitat severity (Figure 2), the more covariant relationship among these traits each other would be inferred. However, as herbaceous species did not demonstrate significant convergent patterns for most traits at more stressful habitats (Figure 1, right), we cannot infer the pattern that trait covariance might present along habitat severity. Our Pearson analysis results revealed the difference between the community and woody species and herbaceous species. Significant correlations among leaf morphological traits are not surprising. LA, SLA, and LDMC are directly related to each other. We found dissimilar patterns of trait-trait relationships between the community and herbaceous species. However, the trait-trait relationships between the community and woody species were very similar. One study explored the relationship between SM and plant height, concluding that plants with more biomass can afford to allocate more energy to seed development, thus producing larger seeds (Thuiller et al., 2004). These results illustrate that the SM of herbaceous species is not covariant with Hmax, suggesting that there are unique strategies of dispersal for herbaceous species compared to the community and woody species. Other trait-trait relations of herbaceous species comparing with community further highlighted the specificity of herbaceous species. Considering that (1) traits change similarly along a habitat stress gradient across growth forms, and (2) trait-trait relationships exhibit different patterns across growth forms, we conclude that the differing community assembly mechanisms between community and woody species and herbaceous species are likely caused by different trait combinations that are filtered by the environment.

## Author contributions

JX and MY: Conceived and designed the experiments; YFC and MW: Analyzed the data; HD: Wrote the paper; YG: Conducted field work; YC, TL, and LZ: Performed the experiments and collected the data; CZ: Helped completing the manuscript.

### Conflict of interest statement

The authors declare that the research was conducted in the absence of any commercial or financial relationships that could be construed as a potential conflict of interest.

## References

[B1] Ackerly D. D. Cornwell W. K. (2007). A trait-based approach to community assembly: partitioning of species trait values into within- and among-community components. Ecol. Lett. 10, 135–145. 10.1111/j.1461-0248.2006.01006.x 17257101

[B2] Borchardt P. Oldeland J. Ponsens J. Schickhoff U. (2013). Plant functional traits match grazing gradient and vegetation patterns on mountain pastures in sw Kyrgyzstan. Phytocoenologia 43, 171–181. 10.1127/0340-269X/2013/0043-0542

[B3] Brouillette L. C. Mason C. M. Shirk R. Y. Donovan L. A. (2014). Adaptive differentiation of traits related to resource use in a desert annual along a resource gradient. New Phytol. 201, 1316–1327. 10.1111/nph.12628 24325125

[B4] Cavender-Bares J. Keen A. Miles B. (2006). Phylogenetic structure of Floridian plant communities depends on taxonomic and spatial scale. Ecology 87, S109–S122. 10.1890/0012-9658(2006)87[109:PSOFPC]2.0.CO;2 16922307

[B5] Chapin F. S. III. Sala O. E. Burke I. C. Grime J. P. Hooper D. U. Lauenroth W. K. (1998). Ecosystem consequences of changing biodiversity. Bioscience 48, 45–52. 10.2307/1313227

[B6] Cheng J. Chu P. Chen D. Bai Y. (2016). Functional correlations between specific leaf area and specific root length along a regional environmental gradient in inner Mongolia grasslands. Funct. Ecol. 658, 259–280. 10.1111/1365-2435.12569

[B7] Cordell S. Handley L. L. (1999). Allocation of nitrogen and carbon in leaves of metrosideros polymorpha regulates carboxylation capacity and δ13c along an altitudinal gradient. Funct. Ecol. 13, 811–818. 10.1046/j.1365-2435.1999.00381.x

[B8] Cornelissen J. H. (1999). A triangular relationship between leaf size and seed size among woody species: allometry, ontogeny, ecology and taxonomy. Oecologia 118, 248–255. 10.1007/s004420050725 28307701

[B9] Cornelissen J. H. C. Lavorel S. B. Garnier E. B. Díaz S. M. Buchmann N. Gurvich D. E. C. (2003). Handbook of protocols for standardised and easy measurement of plant functional traits worldwide. Austr. J. Bot. 51, 335–380. 10.1071/BT02124

[B10] Cornwell W. Ackerly D. (2009). Community assembly and shifts in plant trait distributions across an environmental gradient in coastal California. Ecol. Monogr. 79, 109–126. 10.1890/07-1134.1

[B11] Cunningham S. A. Summerhayes B. Westoby M. (1999). Evolutionary divergences in leaf structure and chemistry, comparing rainfall and soil nutrient gradients. Ecol. Monogr. 69, 569–588. 10.1890/0012-9615(1999)069[0569:EDILSA]2.0.CO;2

[B12] de Bello F. Leps J. Sebastià M. T. (2005). Predictive value of plant traits to grazing along a climatic gradient in the mediterranean. J. Appl. Ecol. 42, 824–833. 10.1111/j.1365-2664.2005.01079.x

[B13] de Bello F. Leps J. Sebastià M. T. (2006). Variations in species and functional plant diversity along climatic and grazing gradients. Ecography 29, 801–810. 10.1111/j.2006.0906-7590.04683.x

[B14] Díaz S. Cabido M. (1997). Plant functional types and ecosystem function in relation to global change. J. Veg. Sci. 8, 463–474. 10.1111/j.1654-1103.1997.tb00842.x

[B15] Díaz S. Cabido M. Casanoves F. (1998). Plant functional traits and environmental filters at a regional scale. J. Veg. Sci. 9, 113–122. 10.2307/3237229

[B16] Díaz S. Cabido M. Zak M. Carretero E. M. Araníbar J. (1999). Plant functional traits, ecosystem structure and land-use history along a climatic gradient in central-western Argentina. J. Veg. Sci. 10, 651–660. 10.2307/3237080

[B17] Díaz S. Lavorel S. de Bello F. Quétier F. Grigulis K. Robson T. M. (2007). Incorporating plant functional diversity effects in ecosystem service assessments. Proc. Natl. Acad. Sci. U.S.A. 104, 20684–20689. 10.1073/pnas.0704716104 18093933 PMC2410063

[B18] Dwyer J. M. Laughlin D. C. (2017). Constraints on trait combinations explain climatic drivers of biodiversity: the importance of trait covariance in community assembly. Ecol. Lett. 20, 872–888. 10.1111/ele.12781 28510261

[B19] Fahn A. Cutler D. F. (1992). Xerophytes Berlin: Gebruder Borntraeger.

[B20] Fonseca C. R. Overton J. M. Collins B. Westoby M. (2000). Shifts in trait-combinations along rainfall and phosphorus gradients. J. Ecol. 88, 964–977. 10.1046/j.1365-2745.2000.00506.x

[B21] Fraser L. H. Garris H. W. Carlyle C. N. (2016). Predicting plant trait similarity along environmental gradients. Plant Ecol. 217, 1297–1306. 10.1007/s11258-016-0628-3

[B22] Fynn R. W. S. Kirkman K. P. (2005). Plant strategies and trait trade-offs influence trends in competitive ability along gradients of soil fertility and disturbance. J. Ecol. 93, 384–394. 10.1111/j.0022-0477.2005.00993.x

[B24] Garnier E. Cortez J. Billès G. Navas M. L. Roumet C. Debussche M. (2004). Plant functional markers capture ecosystem properties during secondary succession. Ecology 85, 2630–2637. 10.1890/03-0799

[B23] Garnier E. Navas M. L. (2012). A trait-based approach to comparative functional plant ecology: concepts, methods and applications for agroecology. A review. Agron. Sustain. Dev. 32, 365–399. 10.1007/s13593-011-0036-y

[B25] Götzenberger L. de Bello F. Bråthen K. A. Davison J. Dubuis A. Guisan A.. (2012). Ecological assembly rules in plant communities—approaches, patterns and prospects. Biol. Rev. 87, 111–127. 10.1111/j.1469-185X.2011.00187.x 21692965

[B26] Grabherr G. Gottfried M. Paull H. (1994). Climate effects on mountain plants. Nature 369, 448–448. 10.1038/369448a0 23320303

[B27] Grime J. P. (2006). Trait convergence and trait divergence in herbaceous plant communities: mechanisms and consequences. J. Veg. Sci. 17, 255–260. 10.1111/j.1654-1103.2006.tb02444.x

[B28] Guittar J. Goldberg D. Klanderud K. Telford R. J. Vandvik V. (2016). Can trait patterns along gradients predict plant community responses to climate change? Ecology 97, 2791–2801. 10.1002/ecy.1500 27859101

[B29] Hadley J. L. Smith W. K. (1986). Wind effects on needles of timberline conifers: seasonal influence on mortality. Ecology 67, 12–19. 10.2307/1938498

[B30] Hulshof C. M. Violle C. Spasojevic M. J. McGill B. Damschen E. Harrison S. (2013). Intra-specific and inter-specific variation in specific leaf area reveal the importance of abiotic and biotic drivers of species diversity across elevation and latitude. J. Veg. Sci. 24, 921–931. 10.1111/jvs.12041

[B31] Joshi C. (2013). Inferential Tests and Modelling of Functional Trait Convergence along Environmental Gradients. International Statistical Institute

[B32] Keddy P. A. (1992). Assembly and response rules: two goals for predictive community ecology. J. Veg. Sci. 3, 157–164. 10.2307/3235676

[B33] Kluge J. Kessler M. (2011). Phylogenetic diversity, trait diversity and niches: species assembly of ferns along a tropical elevational gradient. J. Biogeogr. 38, 394–405. 10.1111/j.1365-2699.2010.02433.x

[B34] Körner C. (1999). Alpine plants: stressed or adapted?, in Physiological Plant Ecology, eds Press M. C. Scholes J. D. Barker M. G. (Oxford, UK: Blackwell), 297–312.

[B35] Kraft N. J. B. Ackerly D. D. (2010). Functional trait and phylogenetic tests of community assembly across spatial scales in an amazonian forest. Ecol. Monogr. 80, 401–422. 10.1890/09-1672.1

[B36] Kraft N. J. B. Valencia R. Ackerly D. D. (2009). Functional traits and niche-based tree community assembly in an amazonian forest. Science. 324, 580–582. 10.1126/science.1169885 18948539

[B37] Lamanna C. Blonder B. Violle C. Kraft N. J. B. Sandel B. Šímová I.. (2014). Functional trait space and the latitudinal diversity gradient. Proc. Natl. Acad. Sci. U.S.A. 111, 13745–13750. 10.1073/pnas.1317722111 25225365 PMC4183280

[B38] Lambrecht S. C. Dawson T. E. (2007). Correlated variation of floral and leaf traits along a moisture availability gradient. Oecologia 151, 574–583. 10.1007/s00442-006-0617-7 17180373

[B39] Landolt E. Bäumler B. Erhardt A. Hegg O. Klötzli F. Lämmler W. (2010). Flora Indicativa - Ecological Inicator Values and Biological Attributes of the Flora of Switzerland and the Alps: Ökologische Zeigerwerte und Biologische Kennzeichen zur Flora der Schweiz und der Alpen. Bern: Haupt.

[B40] Laughlin D. C. (2014). The intrinsic dimensionality of plant traits and its relevance to community assembly. J Ecol. 102, 186–193. 10.1111/1365-2745.12187

[B41] Laughlin D. C. Messier J. (2015). Fitness of multidimensional phenotypes in dynamic adaptive landscapes. Trends in Ecology & Evolution 30:487–496. 10.1016/j.tree.2015.06.003 26122484

[B42] Lavorel S. Díaz S. Cornelissen J. H. C. (2007). Plant functional types: are we getting any closer to the Holy Grail?, in Terrestrial Ecosystems in a Changing World, eds Canadell J. Pataki Deds L. F. (Berlin: Springer-Verlag), 149–164. 10.1007/978-3-540-32730-1_13

[B43] Lawson A. M. Weir J. T. (2014). Latitudinal gradients in climatic-niche evolution accelerate trait evolution at high latitudes. Ecol. Lett. 17, 1427–1436. 10.1111/ele.12346 25168260

[B44] Luo Y. Widmer A. Karrenberg S. (2014). The roles of genetic drift and natural selection in quantitative trait divergence along an altitudinal gradient in *Arabidopsis thaliana*. Heredity 114, 220–228. 10.1038/hdy.2014.89 25293874 PMC4815633

[B45] MacArthur R. H. Wilson E. O. (1967). The Theory of Island Biography. Princeton, NJ.

[B46] Maharjan S. K. Poorter L. Holmgren M. Bongers F. Wieringa J. J. Hawthorne W. D. (2011). Plant functional traits and the distribution of west African rain forest trees along the rainfall gradient. Biotropica, 43, 552–561. 10.1111/j.1744-7429.2010.00747.x

[B48] Marteinsdóttir B. Eriksson O. (2014). Plant community assembly in semi-natural grasslands and ex-arable fields: a trait-based approach. J. Veg. Sci. 25, 77–87. 10.1111/jvs.12058

[B47] Mason N. W. H. de Bello F. (2013). Functional diversity: a tool for answering challenging ecological questions. J. Vegetat. Sci. 24, 777–780. 10.1111/jvs.12097

[B49] Mason N. W. H. Richardson S. J. Peltzer D. A. de Bello F. Wardle D. A. Allen R. B. (2012). Changes in coexistence mechanisms along a long-term soil chronosequence revealed by functional trait diversity. J. Ecol. 100, 678–689. 10.1111/j.1365-2745.2012.01965.x

[B50] May F. Giladi I. Ristow M. Ziv Y. Jeltsch F. (2013). Plant functional traits and community assembly along interacting gradients of productivity and fragmentation. Perspect. Plant EcolEvolut. Syst. 15, 304–318. 10.1016/j.ppees.2013.08.002

[B51] Mcgill B. J. Enquist B. J. Weiher E. Westoby M. (2006). Rebuilding community ecology from functional traits. Trends Ecol. Evol. 21, 178–185. 10.1016/j.tree.2006.02.002 16701083

[B52] McIntyre S. Lavorel S. Landsberg J. Forbes T. D. A. (1999). Disturbance response in vegetation – towards a global perspective on functional traits. J. Veg. Sci. 10, 621–630. 10.2307/3237077

[B53] Meng T. T. Ni J. Harrison S. P. (2009). Plant morphometric traits and climate gradients in northern china: a meta-analysis using quadrat and flora data. Ann. Bot. 104, 1217–1229. 10.1093/aob/mcp230 19805404 PMC2766212

[B54] Meng T. T. Wang H. Harrison S. P. Prentice I. C. Ni J. Wang G. (2015). Responses of leaf traits to climatic gradients: adaptive variation versus compositional shifts. Biogeosciences, 12 1–14. 10.5194/bg-12-5339-2015

[B55] Oyarzabal M. Paruelo J. M. Pino F. D. Oesterheld M. Lauenroth W. K. (2008). Trait differences between grass species along a climatic gradient in south and north America. J. Veg. Sci. 19, 183–192. 10.3170/2007-8-18349

[B56] Pappas C. Fatichi S. Burlando P. (2016). Modeling terrestrial carbon and water dynamics across climatic gradients: does plant trait diversity matter? New Phytol. 59, 137–151. 10.1111/nph.13590 26389742

[B57] Pérez-Harguindeguy N. Garnier E. Lavorel S. Poorter H. Jaureguiberry P. Bret-Harte M. S. (2013). New handbook for standardised measurement of plant functional traits worldwide. Austr. J. Bot. 61, 167–234. 10.1071/BT12225

[B58] Pottier J. Dubuis A. Pellissier L. Maiorano L. Rossier L. Randin C. F. (2012). The accuracy of plant assemblage prediction from species distribution models varies along environmental gradients. Glob. Ecol. Biogeogr. 22, 52–63. 10.1111/j.1466-8238.2012.00790.x

[B59] Purcell A. (2016). Functional Trait Variation along a Hydrological Gradient and Trait-Based Predictions of the Composition of a Wetland Plant Community. A Thesis Submitted in Partial Fulfillment of the Requirements for the degree of Masters of Science, The University of Waikato.

[B60] Qi J. Ma K. Zhang Y. (2009). Leaf-trait relationships of quercus liaotungensis, along an altitudinal gradient in dongling mountain, beijing. Ecol. Res. 24, 1243–1250. 10.1007/s11284-009-0608-3

[B61] Schwinning S. Weiner J. (1998). Mechanisms determining the degree of size asymmetry in competition among plants. Oecologia 113, 447–455. 10.1007/s004420050397 28308024

[B62] Shipley B. (2015). Describing, explaining and predicting community assembly: a convincing trait-based case study. J. Veg. Sci. 26, 615–616. 10.1111/jvs.12294

[B63] Smith B. Wilson J. B. (1994). Vegetation texture as an approach to community structure: community-level convergence in a new Zealand temperate rainforest. N. Zeal. J. Ecol. 18, 41–50.

[B64] Southwood T. R. E. (1988). Tactics, strategies and templets. Oikos 52, 3–18. 10.2307/3565974

[B65] Spasojevic M. J. Suding K. N. (2012). Inferring community assembly mechanisms from functional diversity patterns: the importance of multiple assembly processes. J. Ecol. 100, 652–661. 10.1111/j.1365-2745.2011.01945.x

[B66] Stubbs W. J. Wilson J. B. (2004). Evidence for limiting similarity in a sand dune community. J. Ecol. 92, 557–567. 10.1111/j.0022-0477.2004.00898.x

[B67] Tang Z. Y. Fang J. Y. (2004). Patterns of woody plant species diversity along environmental gradients on Mt.Taibai, Qinling Mountains. Chin. Biodiversity, 12, 115–122

[B68] Thuiller W. Lavorel S. Midgley G. Lavergne S. Rebelo T. (2004). Relating plant traits and species distributions along bioclimatic gradients for 88 leucadendron taxa. Ecology, 85, 1688–1699. 10.1890/03-0148

[B69] Tranquillini W. (1964). The physiology of plants at high altitudes. Plant Biol. 15, 345–362. 10.1146/annurev.pp.15.060164.002021

[B70] Violle C. Bonis A. Plantegenest M. Cudennec C. Damgaard C. Marion B. (2011). Plant functional traits capture species richness variations along a flooding gradient. Oikos 120, 389–398. 10.1111/j.1600-0706.2010.18525.x

[B71] Violle C. Lecoeur J. Navas M. L. (2007). How relevant are instantaneous measurements for assessing resource depletion under plant cover? A test on light and soil water availability in 18 herbaceous communities. Funct. Ecol. 21, 185–190. 10.1111/j.1365-2435.2006.01241.x

[B72] Vittoz P. Bodin J. Ungricht S. Burga C. A. Walther G. R. (2008). One century of vegetation change on Isla Persa, a nunatak in the Bernina massif in the Swiss Alps. J. Vegetat. Sci. 19, 671–680. 10.3170/2008-8-18434

[B73] Walther G. R. Beißner S. Burga C. A. (2009). Trends in upward shift of alpine plants. J. Veg. Sci. 16, 541–548. 10.1111/j.1654-1103.2005.tb02394.x

[B74] Webb C. O. (2000). Exploring the phylogenetic structure of ecological communities: an example for rain forest trees. Am. Nat. 156, 145–155. 10.1086/303378 10856198

[B75] Weiher E. Keddy P. A. (1995). Assembly rules, null models, and trait dispersion: new questions from old patterns. Oikos 74, 159–164. 10.2307/3545686

[B76] Weiher E. Keddy P. A. (1998). Community assembly rules, morphological dispersion, and the coexistence of plant species. Oikos 81, 309–322. 10.2307/3547051

[B77] Westoby M. (1998). A leaf-height-seed (lhs) plant ecology strategy scheme. Plant Soil 199, 213–227. 10.1023/A:1004327224729

[B78] Westoby M. Wright I. J. (2002). Plant ecological strategies: some leading dimensions of variation between species. Ecol. Evol. Syst. 33, 125–159. 10.1146/annurev.ecolsys.33.010802.150452

[B79] Woodward F. I. Cramer W. (1996). Plant functional types and climatic change: introduction. J. Veg. Sci. 7, 306–308. 10.1111/j.1654-1103.1996.tb00489.x

[B80] Woodward F. I. Diament A. D. (1991). Functional approaches to predicting the ecological effects of global change. Funct. Ecol. 5, 202–212. 10.2307/2389258

[B81] Wright I. J. Reich P. B. Westoby M. Ackerly D. D. Baruch Z. Bongers F.. (2004). The worldwide leaf economics spectrum. Nature 428, 821–827. 10.1038/nature02403 15103368

[B82] Xu J. Chen Y. Zhang L. Chai Y. Wang M. Guo Y.. (2017). Using phylogeny and functional traits for assessing community assembly along environmental gradients: a deterministic process driven by elevation. Ecol. Evol. 7, 5056–5069. 10.1002/ece3.3068 28770046 PMC5528205

[B83] Yablon E. (2013). Functional Traits, Environmental Gradients and Community Assembly in a Temperate Forest. Senior Honors thesis, Washington University in St. Louis.

[B84] Yan B. Zhang J. Liu Y. Li Z. Huang X. Yang W. (2012). Trait assembly of woody plants in communities across sub-alpine gradients: identifying the role of limiting similarity. J. Veg. Sci. 23, 698–708. 10.1111/j.1654-1103.2011.01384.x

[B85] Zhu Z. C. (1981). The regulation and characteristic of dominant type of forest in Taibai Mountain, Qinling Mountains. Shaanxi Forest Sci. Technol. 5, 29–39.

