# Supplementary material for: Shifts in Plant Community Assembly Processes across Growth Forms along a Habitat Severity Gradient: A Test of the Plant Functional Trait Approach
Source: Front Plant Sci. 2018 Feb 15;9:180. doi: 10.3389/fpls.2018.00180 (PMC5818416; doi:10.3389/fpls.2018.00180)
Supplement: Supplementary file 1 [file DataSheet1.docx]

**Appendix Table 1** PC1 loadings of community, woody species and herbaceous species mean weighted trait values. The trait which significantly correlated with PC1 axes are highlighted in bold.

| Traits | PC1 loading of community | PC1 loading of woody species | PC1 loading of herbaceous species |
| --- | --- | --- | --- |
| LA | **0.843** | **0.847** | **0.679** |
| SLA | **0.814** | **0.804** | **0.821** |
| LDMC | **-0.787** | **-0.756** | **-0.819** |
| Hmax | **0.641** | **0.449** | 0.125 |
| LNC | **0.443** | **0.572** | 0.064 |
| LCC | **-0.74** | **-0.554** | **-0.762** |
| C:N | **-0.717** | **-0.768** | **-0.349** |
| SM | **0.888** | **0.899** | **0.651** |

**Appendix Table 2** Results of variance partitioning among community, woody species and herbaceous species level. Environment factor codes are as follows: Elevation, elevation of plot; SWC, soil water content; TN, soil total nitrogen content; AN, soil ammonium nitrogen content; NN, soil nitrate nitrogen content; RAP, soil rapid available phosphorus content; pH, soil pH value; Slope, slope of plot; WCD, woody species coverage degree.

|  | Community | Woody species | Herbaceous species |
| --- | --- | --- | --- |
| pH | 4.548 | 4.264 | 4.080 |
| SWC | 16.006 | 17.937 | 13.682 |
| NN | 1.339 | 1.159 | 1.922 |
| AN | 3.893 | 4.202 | 3.878 |
| TN | 4.088 | 4.298 | 4.180 |
| RAP | 5.362 | 6.145 | 5.335 |
| Slope | 10.580 | 11.971 | 10.758 |
| WCD | 1.316 | 1.442 | 1.366 |
| MAT | 26.107 | 23.931 | 26.921 |
| RH | 26.762 | 24.651 | 27.878 |

**Appendix Table 3** The Pearson correlation coefficient of HV and elevation. HV means habitat-severity values. They have a significant correlation each other (highlight in bold).

|  | HV of community | HV of woody species | HV of herbaceous species |
| --- | --- | --- | --- |
| HV of woody species | **0.992** |  |  |
| HV of herbaceous species | **0.996** | **0.981** |  |
| Elevation | **0.930** | **0.978** | **0.950** |

**
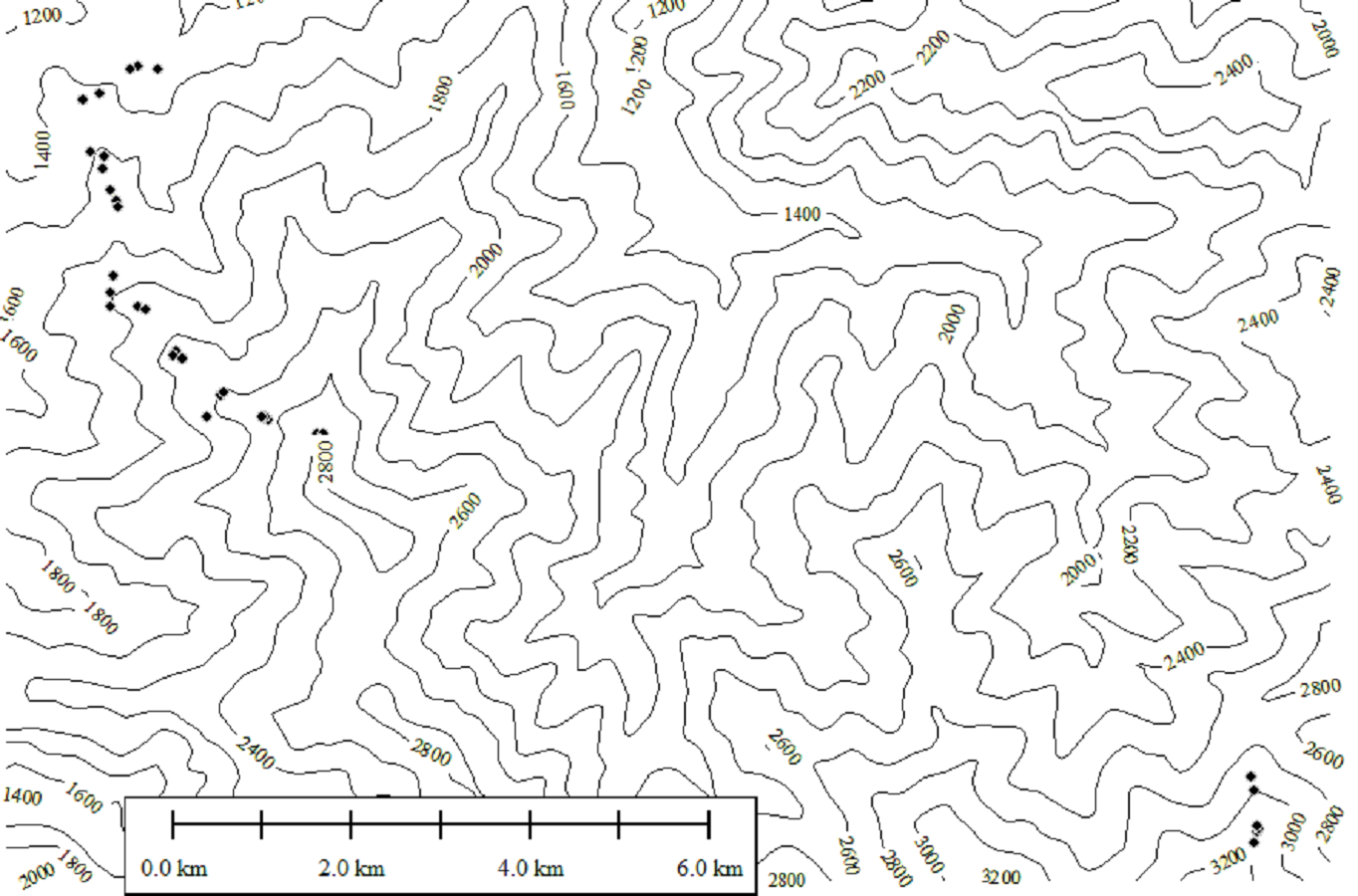
**

**Appendix Figure 1** Sampling maps. The solid circle represents sampling plots. The curve in graph represents isohypse line. The number under curve represents elevation.
